# A Case of a Fibrous Omphalomesenteric Duct Remnant Causing an Intestinal Obstruction in an Adult

**DOI:** 10.15388/Amed.2024.31.2.1

**Published:** 2024-12-04

**Authors:** Camille Tonneau, Jerome Herve, Benjamin Nebbot, Olivier Cappeliez, Sanjiva Pather, Thomas Saliba

**Affiliations:** 1 Hopital de Braine L’Alleud, Braine L’Alleud, Belgium

**Keywords:** omphalomesenteric duct, abdominal surgery, emergency, adult, occlusion, case report, Raktažodžiai: omfalomezenterinis latakas, pilvo chirurgija, skubi pagalba, suaugęs žmogus, okliuzija, atvejo aprašymas

## Abstract

The omphalomesenteric canal (OMC) is an important embryonic structure that normally regresses during development. OMC remnant persistance is rare and can lead to complications such as small intestinal obstruction. We report the case of an 18-year-old male with flu-like symptoms, abdominal pain, fever, and a positive McBurney sign. A CT scan raised the suspicion of occlusion, prompting surgery, revealing a fibrous band from the umbilicus to the mesocolon around which the right colon and caecum were wrapped. OMC anomalies are generally encountered in children, with a large spectrum of possibilities causing various problems. Diagnosis is challenging, with symptoms often mimicking other conditions, often necessitating surgery to obtain a definitive diagnosis. Intestinal obstruction is a severe complication, necessitating urgent surgical resection. Radiological imaging mainly serves to prompt surgical intervention as it is limited in directly visualizing fibrous bands, with surgery remaining the best way to obtain a diagnosis, as well as allowing concomitant treatment.

## Abstract (it)

Omfalomezenterinis latakas (OMC) yra svarbi embrioninė struktūra, kuri vystymosi metu paprastai regresuoja. OMC liekanų išlieka retai ir tai gali sukelti komplikacijų, pavyzdžiui, plonosios žarnos nepraeinamumą.

*Atvejo aprašymas*. 18 metų vyrui atsirado į gripą panašių simptomų, pilvo skausmas, karščiavimas ir buvo teigiamas McBurney požymis. Atlikus kompiuterinės tomografijos tyrimą kilo įtarimas dėl okliuzijos, todėl buvo atlikta operacija. Jos metu paaiškėjo esanti skaidulinė juosta nuo bambos iki mezokolono, aplink kurią buvo apsivyniojusi dešinioji storoji žarna ir akloji žarna. Paprastai susiduriama su vaikų OMC anomalijomis, o įvairių problemų sukelia didelis spektras galimybių. Diagnozė yra sudėtinga, nes simptomai dažnokai imituoja kitas ligas, todėl dažnai prireikia operacijos galutinei diagnozei nustatyti. Žarnų nepraeinamumas yra sunki komplikacija, prireikia skubios chirurginės rezekcijos. Radiologinis vaizdas dažniausiai padeda paskatinti chirurginį įsikišimą, nes juo galima tiesiogiai vizualizuoti fibrozines juostas, o operacija tebėra geriausias būdas diagnozei nustatyti, taip pat leidžia taikyti gretutinį gydymą.

## Introduction

The omphalomesenteric canal (OMC), also known as the yolk canal, plays a crucial role in embryonic development. During embryogenesis, the OMC connects the primitive intestine to the yolk sac, which is responsible for nourishing the embryo during the early stages of development. This channel allows the transport of nutrients and fluids from the yolk sac to the developing primitive intestine. [1]

As the embryo develops, the OMC undergoes a series of transformations and regresses, eventually disappearing entirely. These changes are essential for normal formation of the gastrointestinal tract in the developing embryo. [1]

In approximately 2 to 3% of the general population this canal persists, partially or in its entirety. [2,3] Small intestine obstruction due to persistent OMC, particularly in adult patients, is extremely rare with very few cases reported in the literature. [4-6] Such a situation requires immediate abdominal surgery with the aim of resecting the remnant of the canal. [7,8]

We report the case of an 18-year-old male who presented to the emergency department with abdominal pain.

## Case report

**Fig 1:**
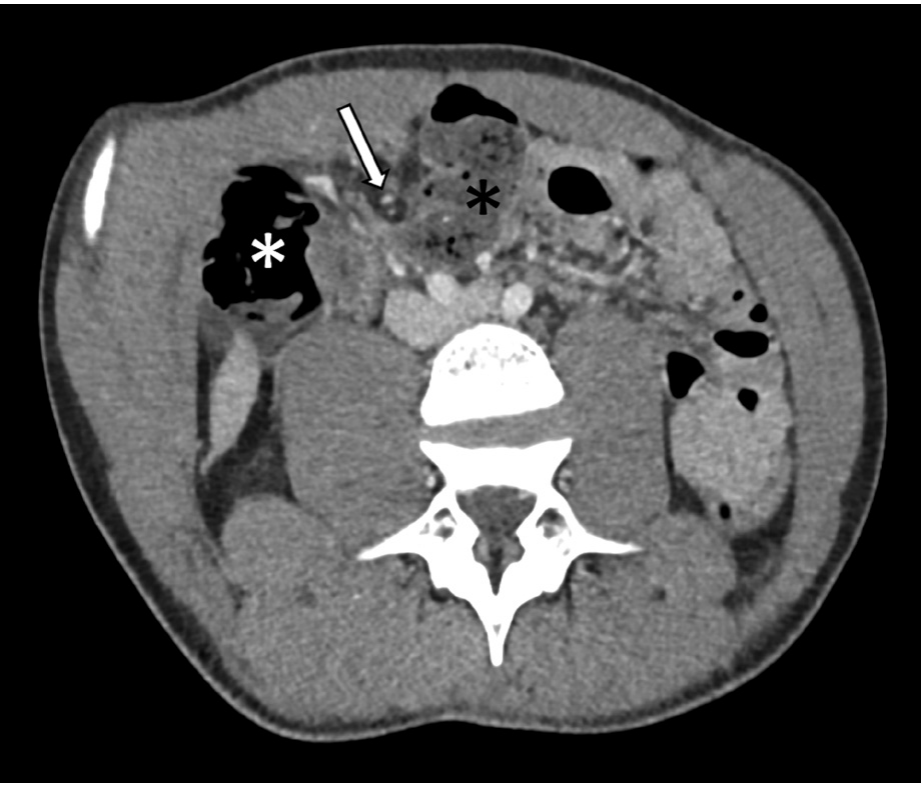
Axial/oblique image from a contrast enhanced CT exam showing feces-like material within the ileum (black star) with a transition point in the right iliac fossa around a fibrous band (arrow). The colon is on the other side of the transition point (white star).

An 18-year-old patient presented to the emergency room with abdominal pain that appeared overnight and was located in the right iliac fossa. The patient had nausea but no vomiting and had been suffering from fever for 1 week accompanied by flu-like symptoms. The clinical examination revealed a supple abdomen but a positive McBurney sign. A blood test was performed, finding neither signs of inflammation, nor a raised white blood cell count. A contrast enhanced CT exam was requested to exclude appendicitis or another acute pathology, revealing what appeared to be feces within the ileum with a transition point in right iliac fossa (Fig 1), accompanied by ascites within the retrovesical pouch. There was no pathological distention of the intestines. The radiologist hypothesized that there may be a subocclusion due to the suspected feces sign, with the lead point being an unseen adhesion and therefore recommended surgical exploration.

**Fig 2:**
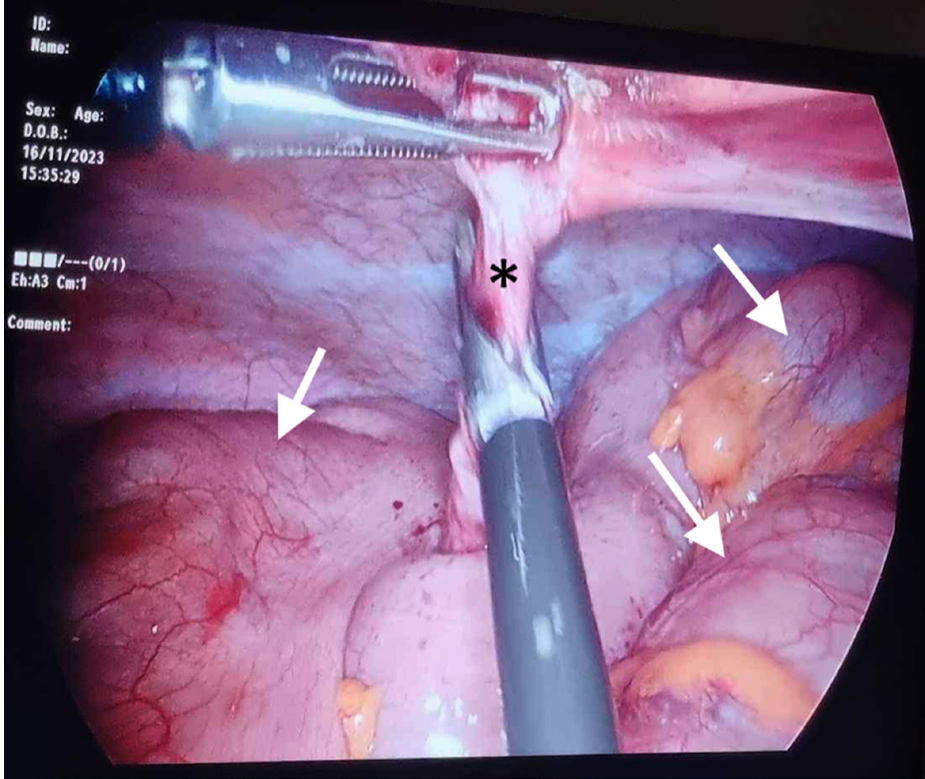
Peroperative celioscopic image showing the intestines (arrows) twisted around a cord (star), corresponding to the fibrous band seen on CT.

With this information, the surgical team decided to perform an exploratory laparoscopy. This procedure revealed a fibrous band starting from the umbilicus and ending at the right mesocolon, corresponding to a fibrous remnant of the OMC. Furthermore, it was found that the right colon and the cecum had wrapped themselves around this fibrous band (Fig 2). The appendix was also seen, being of normal size and appearance.

**Fig 3:**
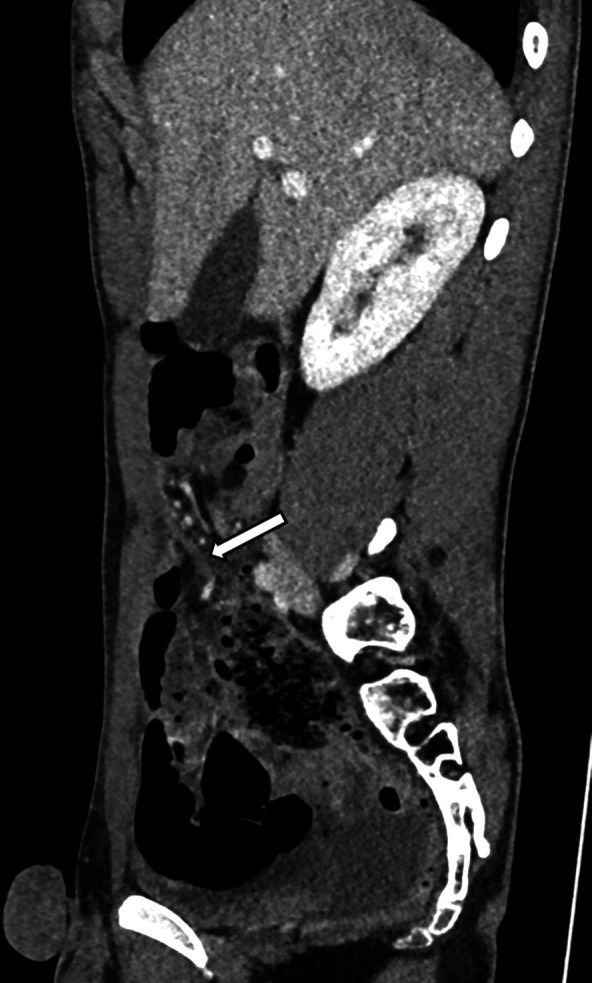
Sagittal image from a contrast-enhanced CT exam showing a fibrous band (arrow) attached to the umbilical area. This band served as the lead point around which the small bowel was wrapped.

Retrospective analysis of the CT scan revealed a fat-density band attached to the umbilicus, corresponding to the mesenteric fat that was wrapped around the fibrous band found during surgery, which provided the lead point for the small bowel volvulus (Fig 3).

The patient’s recovery from the operation was uneventful, with only some pain, associated with the expected colonic dilatation, being reported.

Several fruitless attempts were made to contact the patient and their next of kin by email and phone to obtain consent. Therefore all information and images pertaining to the patient were fully anonymized.

## Discussion

The OMC, or yolk canal, normally ensures communication between the primitive intestine and the yolk sac in the embryo during intrauterine life.^1^ The yolk sac is the seat of heamatopoesis for the early embryo, where large nucleated erythrocytes, megakaryocytes and macrophages emerge from blood islands attached to the yolk sac wall.[9] Furthermore, the yolk sac also hosts primordial germ cells, which exit the embryo during the fourth and fifth week of gestation to reside within the posterior yolk sac, before migrating back into the embryo when the endoderm of the primitive gut performs a lateral fold. [9] The OMC is obliterated and disappears completely between the fifth and ninth week of gestation. [1,2] In approximately 2 to 3% of the general population this canal persists, either partially or in its entirety. [10,11]

This pathology remains rare, most often encountered in the neonatal period. [12] However, our patient was atypical in that he was 18 years old when the anomaly became symptomatic.

When anomalies occur, we can classify them depending on the type and severity of the OMC involution defect. In total involution, the entire canal remains permeable, which is referred to as an omphalo-mesenteric fistula. [13] There are several types of partial involution anomalies. Incomplete involution creates a fibrous band connecting the free edge of the intestine to the deep surface of the umbilicus such as in the case of our patient. [14] Incomplete regression of the yolk canal can result in a fibrous band connecting the posterior surface of the umbilicus to the antemesentric edge of an ileal loop, which can be associated with Meckel’s diverticulum, connecting it to the umbilicus. [15] This adhesion can remain silent, only revealing itself once it is responsible for a mechanical intestinal occlusion. [16] If the involution affects the umbilical segment, it will result in an omphalo-mesenteric sinus tract. If the involution affects the middle segment, then it will become a yolk cyst. [17] If it affects the intestinal segment, it will develop into Meckel’s diverticulum. [18] There are few warning signs of imminent occlusion, and those which exist are nonspecific, making the diagnosis before surgery rare. [7,19] When signs do occur they are often a result of complications. Intestinal obstruction is the most lethal complication of OMC remnants. [2,17]

The most frequent symptoms and signs of patients with small bowel obstruction, although variable, are abdominal pain, vomiting, constipation, abdominal distension, and tenderness. [20] Adhesions, incarcerated hernias, and large bowel cancer constitute the most frequent causes of obstruction, adhesions being the leading cause and accounting for 45%–80%. [4]

In view of the high mortality rate of patients with a prolapse of the ileum (18%), and the strong possibility of intestinal obstruction, patent OMCs should be surgically resected. [8,21] In previous cases described in the literature patients presented with symptoms of obstruction such as stopping of flatus and stools or abdominal distension, however our patient did not present with any of these. [19] There are several case reports of adults with symptoms of occlusion in whom ileal resection was necessary. [4,6] By taking into consideration the diagnosis of a persistent duct in an adult with an acute abdomen without a history of surgery it may be possible to avoid unnecessary intestinal resections.[22] Imaging will be able to provide the diagnosis of intestinal obstruction with a transition point if this exists, although the fibrous bands are generally not seen directly using a CT exam. [4,23] Although rarely directly seen, one account of the identification of a dense fibrous band by CT exists, though in the case of our patient the band was of fatty density and thus was not identified immediately as the cause of the obstruction. [4] A contrast enema CT scan may provide findings which are characteristic but not specific of this condition. [23] As the conclusive diagnosis is not able to be made using radiology, the radiologist’s role is essentially to provide the information which will prompt the surgeon to intervene. Once the surgeons intervene, the diagnosis of an OMC anomaly is generally made peroperatively. Surgery remains the “gold standard” of treatment, and it involves intestinal resection, either by conventional surgery or laparoscopic surgery.

## Conclusion

OMC anomalies are rare outside of the young pediatric population. When they occur, they generally present with symptoms of occlusion. Radiology is of limited use in their detection, its role being limited to providing enough reasons for the surgical team to intervene.

We presented a case of an 18-year-old boy with a case of an OMC fibrous band resulting in an occlusion. The patient was atypical in terms of the age as well as the symptomatology. Although uncommon, this case demonstrates the need to consider OMC anomalies, even in patients who are beyond the age at which anomalies tend to present themselves.
